# A Repeat Case of Cefotetan-Induced Hemolytic Anemia in a Surgical Patient

**DOI:** 10.7759/cureus.30675

**Published:** 2022-10-25

**Authors:** Alayna Gallagher, David Roberts, Raymond Kwok, Terenig Terjanian, Indraneil Mukherjee

**Affiliations:** 1 Internal Medicine, Staten Island University Hospital, Staten Island, USA; 2 College of Medicine, Touro College of Osteopathic Medicine, New York City, USA; 3 Surgery, Staten Island University Hospital, Staten Island, USA; 4 Hematology and Oncology, Staten Island University Hospital, Staten Island, USA

**Keywords:** laparoscopic cholecystectomy (lc), cefotetan, cephalosporin induced hemolytic anemia, prophylactic antibiotic use, drug induced immune hemolytic anemia

## Abstract

Drug-induced hemolytic anemia is a rare hematologic condition in which the immune system creates antibodies against red blood cell antigens in response to a medication exposure. This condition is commonly triggered by antibiotics, nonsteroidal anti-inflammatory drugs, and certain chemotherapies. Here, we describe a patient who experienced a repeat episode of drug-induced hemolytic anemia related to prophylactic cefotetan given before surgery. This case is important for increasing awareness of cephalosporin-induced hemolytic anemia, illustrating the significance of early detection and treatment of the condition, and highlighting the need for careful review of medication history in the surgical setting.

## Introduction

Autoimmune hemolytic anemias (AIHA) are a group of rare hematologic disorders characterized by the breakdown of red blood cells (RBCs) secondary to antibody formation against erythrocyte antigens. AIHA can be further classified into primary and secondary AIHA [1]. Primary AIHA is classified as either warm antibody or cold antibody-mediated, with warm antibody-mediated AIHA being the more common type. Warm AIHA accounts for approximately half of all cases, with a prevalence of one per 75,000 to 80,000 population [1]. Warm AIHA is most often mediated by Immunoglobulin G (IgG) binding to rhesus proteins or glycophorin A-D antigens found on the surface of RBCs. These antibodies maximally bind at higher temperatures close to 37°C. The binding of these antibodies to antigens allows for complement factors to adhere to the surface via the classical complement pathway. The result of complement fixation is extravascular hemolysis [2]. Primary warm AIHA typically presents with an insidious onset of mild anemia occurring over months, and individuals are often asymptomatic. Splenomegaly may occur but is often mild [1]. In severe cases, a physical examination may be remarkable for skin pallor, fever, jaundice, hepatosplenomegaly, hyperpnea, tachycardia, angina, or heart failure [1].

Secondary AIHA is commonly drug-induced or occurs as a manifestation of another disease process [1]. Secondary cold hemolytic anemia is often seen in patients with mycoplasma pneumonia, infectious mononucleosis, and varicella. These are often self-limited processes, lasting less than three weeks [1]. Secondary warm AIHA can be due to hematological malignancies, such as chronic lymphocytic leukemia (CLL) and lymphomas, and it accounts for approximately half of all secondary AIHA cases. Secondary warm AIHA is also commonly associated with systemic lupus erythematosus (SLE) and other autoimmune diseases [1]. Drug-induced immune hemolytic anemia (DIIHA) is commonly triggered by antibiotics such as cephalosporins and penicillin, nonsteroidal anti-inflammatory drugs (NSAIDs), and platinum-based chemotherapies [3]. This group is often classified mechanistically into three models, including the drug-independent model, immune complex model, and hapten-mediated model. In the drug-independent model, the addition of a drug to a patient’s serum will induce the production of autoantibodies against intrinsic red cell antigens. This is commonly associated with drugs such as 𝛂 - methyldopa. The immune complex model is mediated by initial antibody formation to a drug, most commonly quinidines. Upon drug adherence to the RBC surface, the antigen-antibody complex will adsorb to the surface of the RBC and activate the complement cascade. Lastly, the hapten-mediated model occurs when a drug binds to the surface, creating a new epitope that stimulates IgG autoantibody production. This model is often used to describe the method by which penicillin and cephalosporins can induce AIHA [4,5].

The incidence of DIIHA is estimated to be one in 1 million per year, with the most frequently reported drugs, including cephalosporins, diclofenac, oxaliplatin, fludarabine, and rifampin [6]. One study reviewing cases over 10 years reported 17 cases due to ceftriaxone and 37 cases due to other cephalosporins, most commonly cefotetan [7]. Patients suffering from DIIHA often either present acutely within hours of exposure to the drug with severe intravascular hemolysis and symptoms of acute jaundice, dark urine, and back pain or subacutely within weeks to months with typical symptoms of anemia, including fatigue and pallor [6]. Initial workup often shows decreased haptoglobin, elevated reticulocytes, elevated lactate dehydrogenase (LDH), increased unconjugated bilirubin, and a positive direct antiglobulin test (DAT) for IgG and C3. The peripheral blood smear shows schistocytes, spherocytes, and reticulocytes. Management depends on the severity of the disease. Discontinuation of the offending drug is a key component of disease management. Further management strategies include initiation of steroids, fluid resuscitation, RBC transfusion, and IVIG [6]. The prognosis of DIIHA is often good, but it can be fatal in up to 6%-15% of cases involving cephalosporins or NSAIDs [6].

While cephalosporins have been previously reported as a major cause of DIIHA, there is still much to be elucidated regarding clinical variations and optimal treatment strategies. In this report, we present the case of a patient diagnosed with a repeat case of cefotetan-induced hemolytic anemia in the setting of abdominal surgery.

## Case presentation

A 52-year-old male with a past medical history of warm hemolytic anemia in the setting of bariatric sleeve gastrectomy 11 years prior underwent an uncomplicated laparoscopic cholecystectomy for cholecystitis. The patient received 3 grams of cefotetan in the operating room for antimicrobial prophylaxis and was discharged home the same day. On postoperative day five, the patient noticed dark-colored urine and was evaluated by his hematologist. He was found to have acute anemia with a hemoglobin of 8.6 g/dL, decreased from baseline hemoglobin of 15 g/dL. Given his prior history of hemolytic anemia requiring hospitalization, he was advised to present to the emergency department (ED) for further workup and management.

In the ED, the patient was hemodynamically stable with a heart rate of 78 bpm and blood pressure of 120/68 mmHg. He denied shortness of breath or fatigue. He was admitted for further workup (Table 1), which revealed normocytic anemia with hemoglobin of 8.8 g/dL and elevated reticulocyte percentage of 9.8%. Peripheral blood smear showed few nucleated RBCs, many schistocytes, and many reticulocytes per high power field. Additional laboratory studies revealed LDH of 995 U/L, haptoglobin of < 20 mg/dL, and a positive direct antiglobulin IgG test suggesting warm AIHA.

**Table 1:** Patient Lab Results

Test	Result	Reference interval
Hemoglobin (Hb)	8.8 g/dL	14.0-18.0 g/dL
Hematocrit (Hct)	26.6%	42.0-52.0%
Mean corpuscular volume (MCV)	91.1 fL	80.0-94.0 fL
Platelet count	187 K/uL	130-400 K/uL
Reticulocyte percentage	9.8%	0.5-1.5%
Total bilirubin	4.3 mg/dL	0.1-1.2 mg/dL
Indirect bilirubin	3.7 mg/dL	0.2-1.2 mg/dL
Lactate dehydrogenase (LDH)	995 U/L	50-242 U/L
Haptoglobin	< 20 mg/dL	34-200 mg/dL
Direct antiglobulin IgG	positive	negative
Direct antiglobulin complement	negative	negative

The patient was prescribed prednisone 1 mg/kg along with daily folic acid for the treatment of warm AIHA. The patient’s home medications were reviewed. He had not started any new medications, and none of his home medications were associated with AIHA. He further denied a family history of hematologic disorders or any recent diagnosis of malignancy. Evaluation for infectious etiologies such as malaria, babesia, and hepatitis b with peripheral blood smear analysis and hepatitis b surface antigen and core antibody was unremarkable. Workup for autoimmune disorders and lymphoproliferative disorders was also completed and failed to identify the cause of his AIHA. However, the patient endorsed a past episode of autoimmune hemolytic anemia 11 years prior following an uncomplicated sleeve gastrectomy. At that time, he presented to ED with similar symptoms of dark urine, fatigue, and scleral icterus. He was found to be anemic with a markedly elevated indirect bilirubin. Other labs showed an elevated LDH and a positive coombs test. His symptoms and hemoglobin improved with steroids, and he was discharged after one week. Investigation for the etiology was unrevealing, and the cause was believed to be idiopathic warm AIHA.

On the current admission, despite treatment with steroids, the patient’s hemoglobin continued to decrease, and he required one unit of RBC transfusion to maintain hemoglobin above 7 g/dL. He was subsequently started on intravenous immunoglobulin (IVIG) 1g/kg daily for four days and Rituxan 375 mg/m^2^ weekly for four weeks. Given that the two episodes of hemolytic anemia both began after surgery, his surgical records, including the anesthesia chart, were carefully reviewed, which showed that he also received preoperative cefotetan for sleeve gastrectomy antimicrobial prophylaxis. Cefotetan was immediately added to his list of allergies, and he was informed not to receive any cephalosporin again as it was the most likely cause of his warm AIHA. After 11 days in the hospital, his hemoglobin level increased, and he was discharged home with instructions to follow up with his outpatient hematologist. Over the following weeks, his hemoglobin steadily increased, and he reported a resolution of fatigue and weakness. He completed a four-week course of Rituxan and slowly tapered off prednisone over the subsequent months. His hemoglobin was 14.7 g/dL at the six months follow-up visit with his hematologist.

## Discussion

Cefotetan is a second-generation cephalosporin approved by the FDA and recommended as an antimicrobial prophylaxis agent for many intra-abdominal surgeries, including gastric bypass, pancreaticoduodenectomy, open and laparoscopic cholecystectomy, small bowel resection, hernia repair, colorectal surgeries, cesarean delivery, and hysterectomy [8]. The recommended dosing for most adults is 2 grams and is typically given within 60 minutes before incision to prevent surgical site infections [8].

The incidence of cefotetan-induced hematologic abnormalities has been reported in up to 1.4% of exposures with the most severe adverse effect of hemolytic anemia estimated to occur in only a small fraction of these cases. One case series describing four patients with drug-induced hemolytic anemias receiving cefotetan before gastric bypass, gastric banding, and cesarean section surgeries reported that patients presented from home between postoperative days 7-14 with symptoms of weakness, dark urine, jaundice, weight loss, and fever [9]. All four patients were found to have decreased hemoglobin levels ranging from 4.4 to 6.8 g/dL, elevated indirect bilirubin, elevated reticulocyte count, elevated LDH, and a positive direct antiglobulin test [9].

The differential diagnosis for a patient presenting with signs of hemolysis may include transfusion reactions, envenomation reactions, G6PD deficiency, pyruvate kinase deficiency, hemoglobinopathies, infection, hereditary spherocytosis, paroxysmal nocturnal hemoglobinuria, thrombotic thrombocytopenic purpura, hemolytic uremic syndrome, systemic lupus erythematosus, vasculitides and more [10]. Initial workup to differentiate between these various causes of symptomatic anemia, jaundice, and tachycardia should initially include a hemoglobin level, reticulocyte count, lactate dehydrogenase, indirect and direct bilirubin, haptoglobin, direct antiglobulin test, and peripheral blood smear [10].

In patients with a known history of drug-induced hemolytic anemia, the management of perioperative prophylactic antibiotics must be considered to avoid dangerous and potentially fatal outcomes. Retrospective studies like that by Arndt and Garratty suggest that patients are likely to experience increasingly severe cases of hemolytic anemia with each subsequent exposure to the offending drug agent [11]. In these patients, alternative antibiotics must be considered. The American Society of Health-System Pharmacists has published Clinical Practice Guidelines for Antimicrobial Prophylaxis in Surgery along with a joint effort with the Infectious Diseases Society of America, the Surgical Infection Society, and the Society for Healthcare Epidemiology of America [8]. For procedures in which cefotetan is listed as an option for antibiotic prophylaxis, the alternative options are often penicillin or another cephalosporin, such as cefazolin, cefoxitin, or ceftriaxone [8]. This poses a predicament as most of the suggested alternative options for cefotetan are other cephalosporins. In the article by Arndt and Garratty, they explain that while some data suggests there is very little cross-reactivity between cefotetan and ceftriaxone or other cephalosporins, it is usually recommended to avoid all cephalosporins indefinitely [11].

## Conclusions

This case is important for raising awareness of cephalosporin-induced hemolytic anemia and illustrating the significance of early detection and treatment. While DIIHA cases remain quite rare, cephalosporins are known to be common culprits when these cases do occur. Despite this risk, cephalosporins remain a standard antibiotic for antimicrobial prophylaxis in many surgeries. The risk of developing cephalosporin-induced hemolytic anemia likely does not outweigh the cumulative benefits that these antibiotics provide for preventing surgical infections, but caution must be taken in patients with a suspected or known history of autoimmune hemolytic anemia. Though the precise cause of this patient’s initial episode of hemolytic anemia was unclear at the time, it would have been important to closely examine the patient’s surgical history, including anesthesia records to assess all medication exposures before surgery, after surgery, as well as in the operating room. This may have facilitated timely identification of cefotetan as a likely trigger and allowed for consideration of alternative prophylactic antimicrobial agents to avoid recurrence.
